# The Protective Effect of Edaravone on TDP-43 Plus Oxidative Stress-Induced Neurotoxicity in Neuronal Cells: Analysis of Its Neuroprotective Mechanisms Using RNA Sequencing

**DOI:** 10.3390/ph15070842

**Published:** 2022-07-08

**Authors:** Aki Soejima-Kusunoki, Kinya Okada, Ryuta Saito, Kazuhiko Watabe

**Affiliations:** 1 Research Unit of Neuroscience, Mitsubishi Tanabe Pharma Corporation, Yokohama 227-0033, Japan; 2 Discovery Technology Laboratories, Mitsubishi Tanabe Pharma Corporation, Yokohama 227-0033, Japan; okada.kinya@mk.mt-pharma.co.jp (K.O.); saitou.ryuuta@mc.mt-pharma.co.jp (R.S.); 3 Faculty of Health Sciences, Kyorin University, Tokyo 181-8612, Japan; watabe@ks.kyorin-u.ac.jp

**Keywords:** edaravone, TDP-43, transcriptome, amyotrophic lateral sclerosis, Nrf2

## Abstract

Edaravone is a free-radical scavenger drug that was recently approved for the treatment of amyotrophic lateral sclerosis (ALS), a neurodegenerative disease. A pathological hallmark of ALS is the accumulation of ubiquitinated or phosphorylated aggregates of the 43-kDa transactive response DNA binding protein (TDP-43) within the cytoplasm of motor neurons. This study revealed the efficacy of edaravone in preventing neuronal cell death in a TDP-43 proteinopathy model and analyzed the molecular changes associated with the neuroprotection. The viability of the neuronal cells expressing TDP-43 was reduced by oxidative stress, and edaravone (≥10 μmol/L) protected in a concentration-dependent manner against the neurotoxic insult. Differential gene expression analysis revealed changes among pathways related to nuclear erythroid 2-related-factor (Nrf2)-mediated oxidative stress response in cells expressing TDP-43. In edaravone-treated cells expressing TDP-43, significant changes in gene expression were also identified among Nrf2-oxidative response, unfolded protein response, and autophagy pathways. In addition, the expression of genes belonging to phosphatidylinositol metabolism pathways was modified. These findings suggest that the neuroprotective effect of edaravone involves the prevention of TDP-43 misfolding and enhanced clearance of pathological TDP-43 in TDP-43 proteinopathy.

## 1. Introduction

Amyotrophic lateral sclerosis (ALS) is a progressive and fatal neurodegenerative disease that leads to a loss of muscle control due to the degeneration of motor neurons within the brain and spinal cord. The typical clinical presentations of ALS include weakness and paralysis of muscles, speech dysfunction, and respiratory failure followed by death [1,2,3]. An excessive amount of free radicals and reactive oxygen species (ROS) leads to neuronal damage by the peroxidation of unsaturated fatty acids among neuronal cells in patients with ALS [3,4]. ALS is classified into familial ALS and sporadic ALS, and sporadic ALS accounts for almost 90% of ALS patients. Recent studies have shown an extensive accumulation of phosphorylated, ubiquitinated, and truncated transactive response DNA binding protein 43 (TDP-43) in the motor neurons of patients with sporadic ALS, which also have characteristics of TDP-43 proteinopathy [2]. Although the mechanisms leading to neurodegeneration in ALS are still incompletely understood, TDP-43 is likely to represent a central causal factor in sporadic ALS.

Edaravone is a free-radical scavenger that shows antioxidative effects against water-soluble peroxyl radicals and lipid-soluble peroxyl radicals. It also eliminates free radicals, including lipid peroxyl radicals, and peroxynitrite, which is another form of ROS, through its electron-donating properties [5]. Several clinical studies, including phase 2 and two phase 3 trials, have confirmed the safety and efficacy of edaravone in patients with ALS [3,6]. In a phase 3, randomized, double-blind study in patients with early-stage ALS, edaravone significantly reduced the Revised ALS Functional Rating Scale score compared to placebo [6,7,8]. In transgenic mice (G93A) and rats (H46R) with mutations of superoxide dismutase 1 (SOD1), edaravone significantly ameliorated the decline of motor function assessed using the rotarod and grip strength tests while protecting spinal cord motor neurons against degeneration and inhibiting SOD1 protein aggregation [9,10]. In a wobbler mouse model with TDP-43 aggregation and ubiquitination, similar to the clinical pathology of ALS, edaravone significantly suppressed the decline of grip strength and muscle weight, as well as inhibiting spinal cord motor neuron degeneration [11]. Therefore, edaravone can ameliorate declined motor functions and may reduce the progression of ALS symptoms via its protective effects on motor neuron degeneration.

Recently, adult rat neural stem cells from the line 1464R coinfected with adenoviruses expressing wild-type (WT) or C-terminal fragment (CTF) TDP-43 [12,13] were shown to present cytoplasmic aggregates consisting of sarkosyl-insoluble granular materials while containing phosphorylated TDP-43 similar to TDP-43 proteinopathy of neuronal cells. Ethacrynic acid is a glutathione S-transferase inhibitor that increases cellular oxidative stress by depleting glutathione, and it induces TDP-43 C-terminal phosphorylation, insolubilization, C-terminal fragmentation, and cytoplasmic distribution [14]. Although edaravone mitigates high levels of oxidative stress [8], little information regarding its mechanisms of action against motor neuron damages in TDP-43 proteinopathy that mimics ALS pathology is available. In the present study, we examined whether edaravone can prevent cell death in a TDP-43 proteinopathy model system. Secondly, we explored molecular changes associated with neuroprotection.

## 2. Results

### 2.1. The Effect of Edaravone against Cell Death in Rat Neural Stem Cell-Derived Neurons Transduced with Adenoviruses Expressing TDP-43

**Figure 1:**
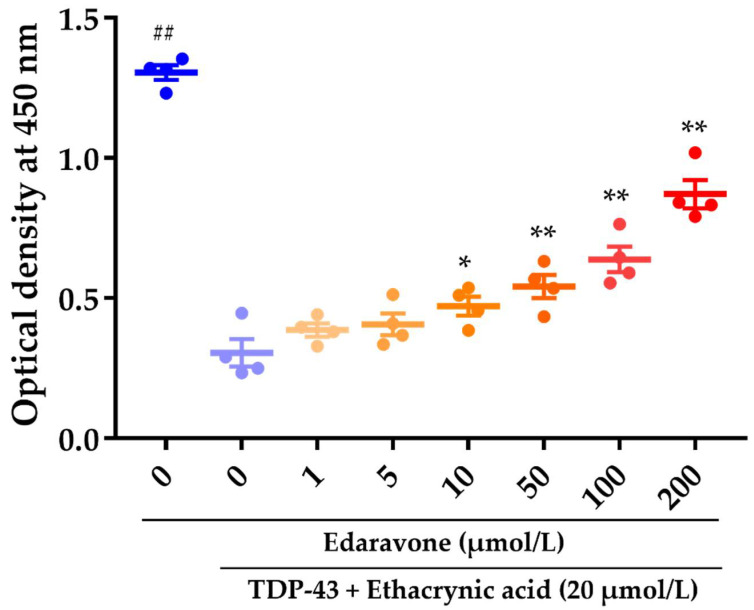
Effects of edaravone against neurotoxicity in rat neural stem cell-derived neurons transduced with adenoviruses expressing wild-type and C-terminal fragment TDP-43 and treated with ethacrynic acid. Neurotoxicity was analyzed as a decrease in cell viability using the CCK-8 assay. Data are expressed as mean ± standard error of the mean (n = 4 wells in 1 experiment). ^##^ *p* < 0.01 compared to the group of TDP-43 plus ethacrynic acid (Student’s *t*-test). * *p* < 0.05, ** *p* < 0.01 compared to the group of TDP-43 plus ethacrynic acid (Williams’ multiple comparison test).

In the present study, we generated a TDP-43 proteinopathy cellular model using adult rat neural stem cells from line 1464R coinfected with adenoviruses expressing WT and CTF TDP-43 [12,13]. The viability of these cells was significantly decreased upon exposure to oxidative stress induced with 20 μmol/L ethacrynic acid (Figure 1). Pretreatment with edaravone 24 h before ethacrynic acid stimulation inhibited cell death associated with TDP-43 proteinopathy at ≥10 μmol/L in a concentration-dependent manner (Figure 1). Simultaneous treatment of edaravone with ethacrynic acid stimulation also showed neuroprotective efficacy (Supplemental Figure S1). In addition, without ethacrynic acid stimulation, the viability of 1464R-derived cells expressing TDP-43 was not different from normal cells at 48 h but decreased after 96 h (Supplemental Figures S2 and S3). Edaravone prevented the reduction in viability of cells expressing TDP-43 under standard conditions without oxidative stress (Supplemental Figure S3).

### 2.2. Transcriptome Analysis

**Figure 2:**
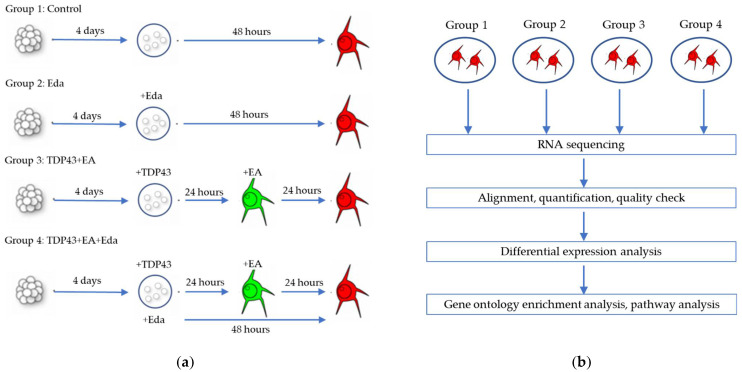
Overview of transcriptome analysis. (**a**) Schematic illustration of experimental design. (**b**) Schematic diagram of RNA sequencing and data analysis (n = 3 in 3 experiments). Eda—Edaravone (50 μmol/L); EA—Ethacrynic acid (20 μmol/L).

From the preliminary analysis, only a small difference was noted in gene expression profiles between TDP-43 overexpressing and non-overexpressing cells (control) cells (Supplemental Table S1). The effect of edaravone on TDP-43-induced neurotoxicity was analyzed with RNA sequencing (Figure 2a) in the following four groups: Control (Group 1), Eda (edaravone, 50 μmol/L) (Group 2), TDP-43 + EA (ethacrynic acid, 20 μmol/L) (Group 3), and TDP-43 + EA (20 μmol/L) + Eda (50 μmol/L) (Group 4). RNA sequencing data obtained from each group were processed using several steps detailed in Figure 2b.

#### 2.2.1. Principal Component Analysis

**Figure 3:**
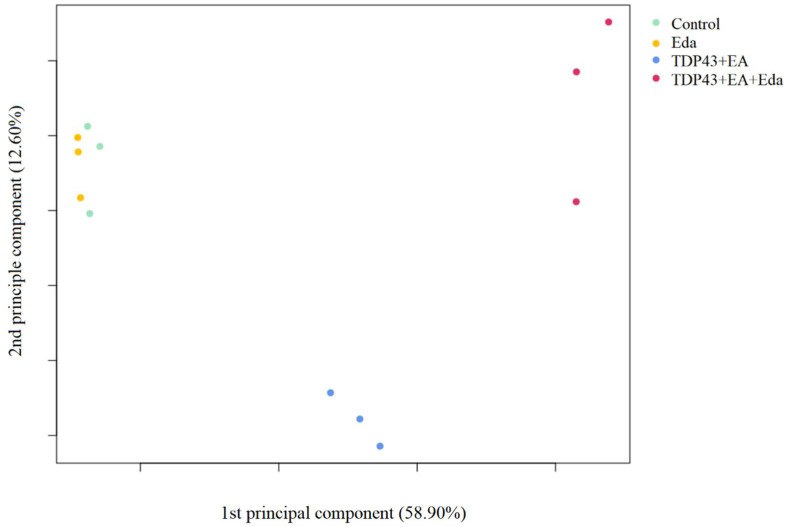
Principal component analysis. Values in parenthesis on the X- and Y-axis titles represent contribution rates. Light green, orange, blue, and red dots represent samples in the Control, Eda (edaravone-treated cells), TDP43 + EA (ethacrynic acid-treated cells expressing TDP-43), and TDP43 + EA + Eda (edaravone- and ethacrynic acid-treated cells expressing TDP-43) groups, respectively. Eda—Edaravone; EA—Ethacrynic acid.

Principal component analysis was performed using expression values from all genes (Figure 3).

There was no significant outlier in any group, and samples from the TDP-43 + EA group (Group 3) were distributed close to each other and were separated from the samples in the Control group (Group 1). Additionally, the samples from the TDP-43 + EA + Eda group (Group 4) showed a similar distribution pattern. The samples from the Control (Group 1) and Eda (Group 2) groups were closer to each other compared to samples from the other groups, implying a relatively similar gene expression among these samples.

#### 2.2.2. Differential Gene Expression Analysis

The criteria for differential gene expression analysis with DESeq2 [15] were set using a *p*-value adjusted with the Benjamini–Hochberg (BH) procedure at ≤0.05 [16] and with an absolute log2 fold change of ≥1. The results of the number of DEGs between the conditions of vehicle, edaravone, or ethacrynic acid treatment in TDP-43 expressed or non-expressed (control) cells are shown in Supplemental Figure S4. To focus on the effect of edaravone, the TDP43 + EA group (Group 3) and the Control group (Group 1) were compared using these criteria in the differential expression analysis. As a result, a total of 1320 genes (853 upregulated and 467 downregulated) were identified as significantly differentially expressed genes (DEGs) (Table 1).

**Figure 4:**
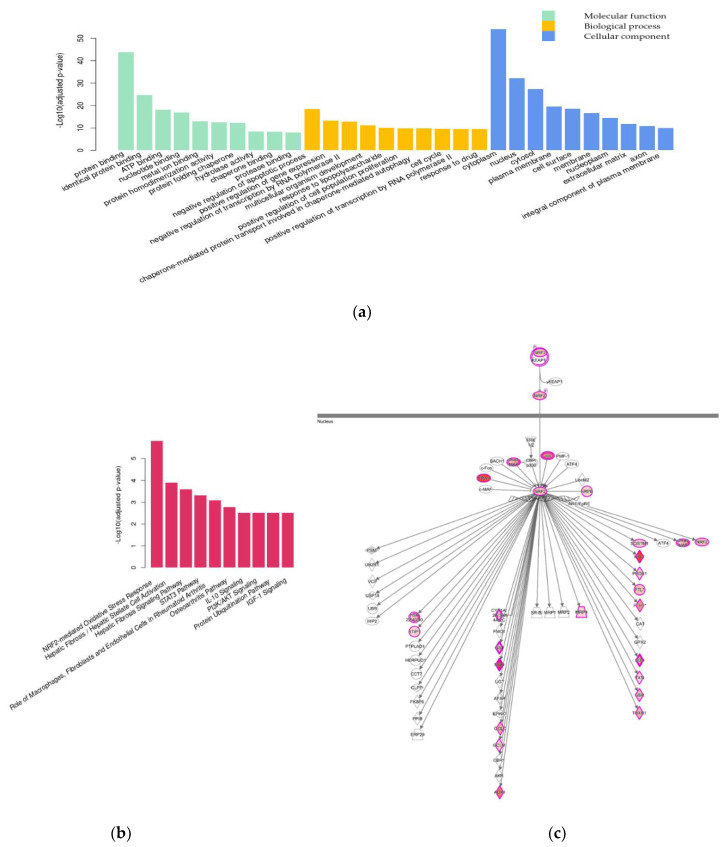
Gene Ontology (GO) enrichment analysis and pathway analysis of differentially expressed genes (DEGs) among differentiated neuronal cells affected by neurotoxicity induced by TDP-43 and ethacrynic acid. (**a**) The top 10 significantly enriched GO terms according to a hypergeometric test *p*-value adjusted with the Benjamini–Hochberg (BH) procedure. Green bars: GO terms in molecular function category; orange bars: GO terms in biological process category; blue bars: GO terms in cellular component category. (**b**) The top 10 significant pathways according to *p*-value adjusted with the BH procedure. (**c**) The illustration of the nuclear erythroid 2-related-factor (Nrf2)-mediated oxidative stress response pathway is generated using the Ingenuity Pathway Analysis software. Red objects represent genes that were upregulated in the neurotoxic condition induced by TDP-43 and ethacrynic acid.

The functional complexity of the DEGs was examined with a Gene Ontology (GO) enrichment analysis [17,18] using a hypergeometric test. Figure 4a shows the 10 most significantly enriched GO terms in each GO category (BH-adjusted *p*-value ≤ 0.05 [16]).

In the molecular function category, GO terms related to binding activity, including protein binding (GO:0005515), metal ion binding (GO:0046872), and identical protein binding (GO:0042802), were enriched in the DEGs. In the biological process category, the enriched DEGs were involved in transcription and apoptosis processes, such as the positive/negative regulation of transcription by RNA polymerase II (GO:0045944; GO:0000122) and negative regulation of apoptosis (GO:0043066). In the cellular component category, the enriched GO terms included cytoplasm (GO:0005737) and nucleus (GO:0005634) compartments.

Pathway analysis was performed, and the pathways associated with the DEGs with BH-adjusted *p*-value ≤ 0.05 [16] are shown in Figure 4b. Significantly enriched pathways in the TDP-43 + EA group (Group 3) compared to the Control group (Group 1) included the nuclear erythroid 2-related-factor (Nrf2)-mediated oxidative stress response pathway, and most genes in the pathway, such as Nrf2 and its related genes, were upregulated in the TDP-43 + EA group (Group 3) (Figure 4c). This finding indicates that oxidative stress is involved in mediating the neurotoxicity induced by the overexpression of TDP-43 and ethacrynic acid in neuronal cells.

**Figure 5:**
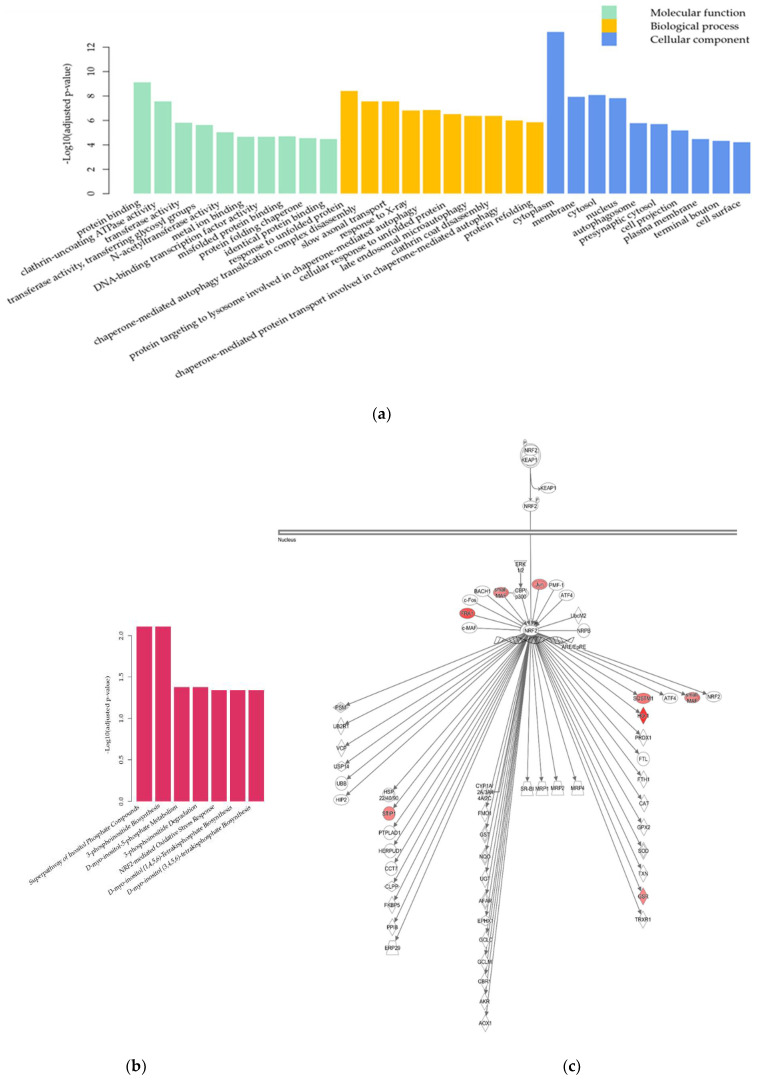
Gene Ontology (GO) enrichment analysis and pathway analysis of differentially expressed genes (DEGs) upon edaravone treatment of differentiated neuronal cells under the neurotoxic condition induced by TDP-43 and ethacrynic acid. (**a**) The top 10 significantly enriched GO terms according to a hypergeometric test *p*-value adjusted with the Benjamini–Hochberg (BH) procedure. Green bars: GO terms in molecular function category; orange bars: GO terms in biological process category; blue bars: GO terms in cellular component category. (**b**) Significant pathways (BH-adjusted *p*-value ≤ 0.05). (**c**) The illustration of the Nrf2-mediated oxidative stress response pathway is generated using the Ingenuity Pathway Analysis software. Red objects represent genes that were upregulated with the addition of edaravone to differentiated neuronal cells under the neurotoxic condition induced by TDP-43 and ethacrynic acid.

In the differential expression analysis comparing the TDP-43 + EA + Eda group (Group 4) to the TDP-43 + EA group (Group 3), a total of 429 DEGs (169 upregulated and 260 downregulated) were identified (Table 1). In the GO category of molecular function, the DEGs were associated with GO terms related to catalytic activity, such as transferase activity (GO:0016740), transferase activity/transferring glycosyl groups (GO:0016757), and binding activity (Figure 5a). In the biological process category, the enriched DEGs were involved in protein folding, e.g., response to unfolded protein (GO:0006986) and protein refolding (GO:0042026), as well as autophagy, e.g., chaperon-mediated autophagy translocation complex disassembly (GO:1904764) and late endosomal microautophagy (GO:0061738). They were located not only in the cytoplasm (GO:0005737) but also in membrane (GO:0016020) compartments.

**Figure 6:**
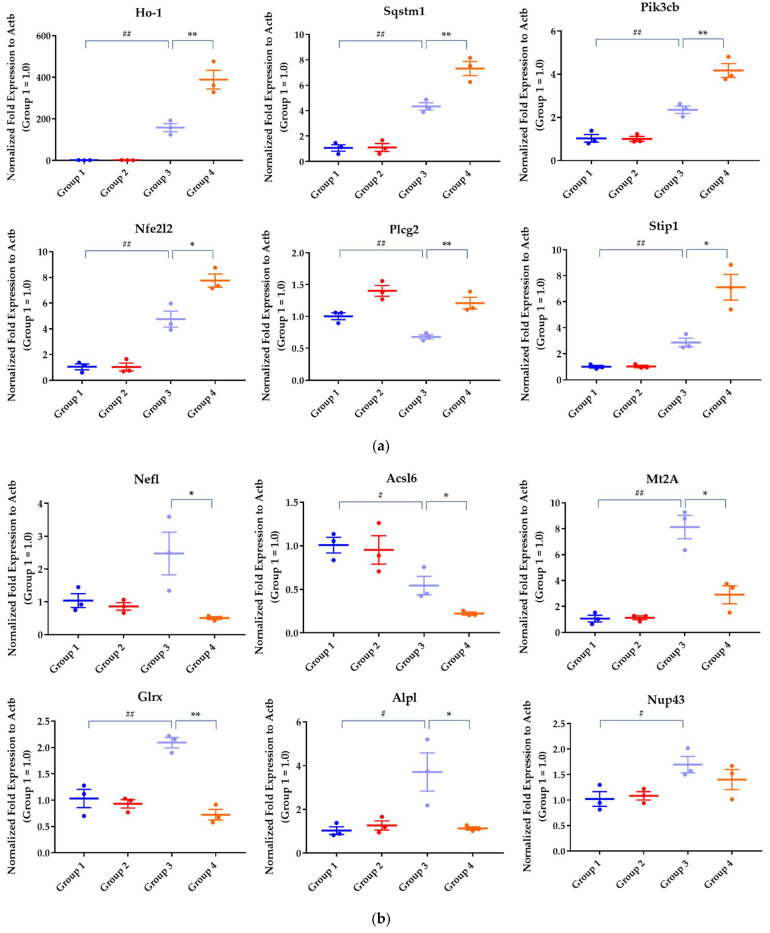
qPCR analysis of representative genes. (**a**) Upregulated and (**b**) downregulated genes upon edaravone treatment. Data are represented as mean ± standard error of the mean (n = 3 in 3 experiments). At first, Group 1 (Control) and Group 3 (TDP-43 + EA) were compared to find the effect of proteinopathy using a Student’s *t*-test # *p* < 0.05, ## *p* < 0.01. Then, the difference between Group 3 and Group 4 (TDP-43 + EA + Eda) was analyzed to find the effect of edaravone on proteinopathy-induced changes using a Student’s *t*-test. * *p* < 0.05, ** *p* < 0.01.

Pathway analysis identified seven significant pathways (Figure 5b), mostly cellular membrane lipid-associated pathways, such as 3-phosphoinositide biosynthesis, and the Nrf2-mediated oxidative stress response pathway. Among the genes in the lipid metabolism pathways, the expression of alkaline phosphatase, biomineralization-associated (ALPL) was downregulated in neuronal cells overexpressing TDP-43 in the presence of ethacrynic acid and upregulated upon edaravone treatment. These cellular membrane lipid-associated pathways were complemented by other pathways significantly differing between the Control (Group 1) and TDP-43 + EA (Group 3) groups, which are listed in Supplemental Table S2. The upregulated DEGs following edaravone treatment included heme oxygenase-1 (Ho-1), glutathione-disulfide reductase (GSR), sequestosome 1 (SQSTM1), and stress-induced phosphoprotein 1 (STIP1) in the Nrf2-mediated oxidative stress response pathway (Figure 5c), supporting the involvement of oxidative stress and autophagy response mechanisms in neuronal cells. Table 2 shows the list of representative genes whose expression was significantly changed by edaravone treatment. The results of quantitative real-time polymerase chain reaction (qPCR) were mostly correlated with DEGs of RNA sequencing (Figure 6). Edaravone upregulated the expression of genes in the Nrf2 pathway, including Ho-1, SQSTM1, STIP-1, nuclear factor, erythroid 2-like 2 (Nfe2l2), and lipid metabolism-related genes (including phosphatidylinositol-4,5-bisphosphate 3-kinase, catalytic subunit beta (Pik3cb), and phospholipase C gamma 2 (Plcg2)) in ethacrynic acid-treated TDP-43 expressed cells (Group 3 vs. Group 4, Figure 6a). On the other hand, genes that were downregulated by edaravone included ALPL, glutaredoxin (Glrx), neurofilament light (Nefl), acyl-CoA synthetase long-chain family member 6 (Acsl6), and metallothionein 2A (Mt2A) (Group 3 vs. Group 4, Figure 6b).

No DEGs significantly differed between the Control (Group 1) and Eda (Group 2) groups, indicating that edaravone did not affect gene expression in 1464R-derived neuronal cells under normal conditions (Table 1). The GO enrichment analysis showed a similar DEG distribution in GO categories following ethacrynic acid treatment in TDP-43 expressed and non-expressed cells (Supplemental Figure S5).

## 3. Discussion

### 3.1. The Effect of Edaravone against Cell Death in Rat Neural Stem Cell-Derived Neurons Transduced with Adenoviruses Expressing TDP-43

In current 1463R differentiated cells, although overexpression of TDP 43 was weakly toxic, oxidative stress triggered by ethacrynic acid extensively increased the neurotoxicity and cell death. Ethacrynic acid depletes intracellular glutathione and induces oxidative stress, similarly to hydrogen peroxide, in NSC-34 cells transduced with TDP-43 [14,19]. Previous studies have shown that oxidative stress resulting from ROS production causes C-terminal phosphorylation, insolubilization, C-terminal fragmentation, and cytoplasmic distribution of TDP-43 in NSC34 cells and primary cortical neurons [14], as well as mislocalization and/or aggregation of TDP-43 in neuronal cells [14,19,20,21,22]. These data suggest that oxidative stress plays a central role in this model. The pathological TDP-43 modification processes in TDP-43 proteinopathy may include cysteine oxidation [23,24] and acetylation [25] of TDP-43, nuclear to cytoplasmic mislocalization of aberrant RNA-binding proteins, increased aggregation, perturbed stress granule dynamics, and cell injury caused by RNA-binding protein localization to mitochondria [26]. Therefore, we consider rat neural stem cell-derived neurons transduced with adenoviruses expressing TDP-43 and undergoing cell death when exposed to ethacrynic acid to represent a reasonable cell model of TDP-43 proteinopathy and cytotoxicity.

We found that the reduction in viability of the TDP-43-expressing 1464R-differentiated neuronal cells was prevented by pre- or co-treatment with edaravone in a concentration-dependent manner in the presence or absence of ethacrynic acid. Considering that edaravone is a free-radical scavenger reducing intracellular ROS in rat primary neuronal, HT-22, and SHSY5Y cells [27,28,29], intracellular oxidative stress was presumed to be mitigated to some extent. The GO enrichment analysis highlighted DEGs of the ROS-related pathways, including positive regulation of reactive oxygen species metabolic process and response to hydrogen peroxide (Supplemental Figure S6). Thus, although the ROS levels were unquantitated in TDP-43 overexpressed 1464R-differentiated neuronal cells, we assumed that ethacrynic acid increased and edaravone reduced the increased ROS levels in this study. On the other hand, in TDP-43 overexpressed cells without oxidative stress by ethacrynic acid, edaravone prevented cell death, suggesting the presence of a mechanism other than the effect on ROS. Furthermore, even though ethacrynic acid induces intracellular oxidative stress by depleting cellular glutathione [30], edaravone did not affect the intracellular levels of cysteine and glutathione in Hepa 1-6 cells [31]. In addition, the 25 kDa CTF can induce cell death through a toxic gain-of-function [32], and cell death without ethacrynic acid stimulation. Therefore, edaravone is presumed to maintain cell viability by suppressing the neurotoxicity pathway resulting from TDP-43 proteinopathy through inhibiting oxidative stress and other mechanisms independent of inhibition of oxidative stress.

### 3.2. Transcriptome Analysis

In differential gene expression analysis, we examined functional clusters of coregulated genes and pathways involved in mediating the effect of edaravone on TDP-43-induced neurotoxicity. In the transcriptome analysis of TDP-43-expressing 1464R cells exposed to ethacrynic acid, we have identified changes in gene expression levels within the pathways of Nrf2-oxidative stress response, unfolded protein response (UPR), autophagy, and phosphoinositide (PI) metabolism, which are discussed below.

#### 3.2.1. Nrf2-Mediated Oxidative Stress Response

Genes involved in the Nrf2-mediated oxidative stress response were significantly enriched in cells transduced with adenoviruses expressing TDP-43 and treated with ethacrynic acid. Under oxidative stress conditions, Nrf2 is phosphorylated and translocated to bind to the antioxidant response element (ARE). As a result of the activation of numerous genes, HO-1 and GSR are upregulated [30,33]. Upregulation of HO-1, which metabolizes heme into Fe^2+^ and biliverdin, prevents the oxidation of proteins and lipid anions and plays a critical role in anti-inflammation, antioxidation, and antiapoptosis processes [3,34]. We assume that the measured gene expression changes among the Nrf2 pathway of TDP-43-expressing 1464R cells exposed to oxidative stress represent the activation of endogenous antioxidant pathways. 

Dysregulation of the Nrf2/ARE signaling pathway were observed in ALS postmortem tissues [34,35], as well as in ALS cellular models with mutant TDP-43 and other ALS-related genes [36,37,38]. As a result, the Nrf2/HO-1 pathway activators could potentially act as a therapeutic approach for future preventive medications in ALS [34,35]. The expression of genes involved in the Nrf2 pathway was upregulated following edaravone treatment in TDP-43-expressing 1464R cells in the presence of ethacrynic acid. It was previously shown that edaravone activates the Nrf2/HO-1 pathway, exerts neuroprotective effects (including a reduction of neurological and cognitive dysfunction), and protects against cell apoptosis in mouse and rat models of various neurological disorders [39,40,41,42]. In the Nrf2/G93A mouse model of ALS pathology, the expression of Nrf2 was accelerated in both spinal cord motor neurons and lower limb muscles during disease progression [43]. In this context, edaravone significantly alleviated the Nrf2 expression and degeneration of motor neurons and muscles [43]. In SH-SY5Y cells of human neuroblastoma, edaravone increased intracellular Nrf2, HO-1, and SOD levels, as well as Nrf2 nuclear translocation upon exposure to amyloid-beta (Aβ)_25–35_ [44]. Therefore, edaravone has the potential to activate the Nrf2/ARE signaling pathway by regulating Nrf2 at the transcriptional and translational levels, as well as at the translocation step [44], under the neurotoxic condition of TDP-43 proteinopathy. 

#### 3.2.2. UPR and Autophagy

Pathway analysis revealed that the expression of STIP1 and SQSTM1 genes was significantly upregulated upon edaravone treatment under the neurotoxic conditions induced by TDP-43 expression and ethacrynic acid. These DEGs are related to UPR and autophagy. STIP1, an intracellular co-chaperone of heat shock proteins (HSPs), facilitates protein transfer from Hsp70 to Hsp90 [45]. Hsp90 and its chaperone allow the mitigation of TDP-43 proteinopathy and toxicity in mammalian cells [46]. Hsp90 has a crucial role recognizing large and intrinsically instable proteins [47]. Protein misfolding is sensed by the endoplasmic reticulum (ER) and initiates the UPR, which is an adaptive process that reduces ER stress to maintain cellular function and viability [47,48]. ER stress is associated with the accumulation of unfolded or misfolded proteins, notably in neurodegenerative diseases and TDP-43 proteinopathy [49,50]. In rodent neuronal cells, edaravone prevented cell death by reducing apoptosis and ER stress [51,52]. These findings suggest that UPR is a pathway mediating the neuroprotective effect of edaravone in TDP-43 proteinopathy.

The DEGs included many genes related to the autophagy pathway. Autophagy is a primary stress response mechanism that delivers protein aggregates and misfolded proteins to lysosomes for degradation [53]. The accumulation of intracellular protein aggregates may result from defective autophagy in neurodegenerative diseases [53]. The SQSTM1 gene encodes the prototype autophagy receptor p62, which facilitates protein degradation through the autophagy system as well as the Nrf2 antioxidant pathway, and presents mutations in patients with ALS [54,55]. In addition, SQSTM1 mutation increases TDP-43-associated stress granule formation [56], and overexpression of p62 decreases TDP-43 aggregation in neuronal cells [57]. However, the direct effects of edaravone on the autophagy pathway remain to be determined. Considering that the autophagy and ER stress UPR pathways are closely associated [58], it will be interesting to study the effects of edaravone on the SQTM1/p62 protein in ALS.

#### 3.2.3. PI Metabolism

Pathway analysis identified seven significant pathways upon edaravone treatment in neuronal cells transduced with adenoviruses expressing TDP-43 and exposed to ethacrynic acid. Most of them were cellular membrane lipid-associated pathways, including inositol phosphate metabolism, as well as 3-PI biosynthesis and degradation. Phosphoinositides (PIPs) play critical roles in regulating myriad cellular processes, and their homeostasis is tightly controlled by numerous inositol kinases and phosphatases [59]. Mutations in PI modulating enzyme genes are largely associated with neurological disorders [59]. For example, FIG4 is a phosphatidylinositol 3,5-bisphosphate-specific 5-phosphatase presenting heterozygous mutations in association with ALS [60].

Among the identified genes belonging to the PI metabolism pathways, the expression of ALPL was downregulated in neuronal cells with TDP-43 overexpression and further upregulated by their treatment with edaravone. The ALPL gene encodes tissue nonspecific isoenzyme of alkaline phosphatase (TNAP) [61], which may mediate axonal growth [61], and emerges as a potential biomarker of disease progression in ALS [62]. Therefore, further investigations of edaravone effects on PI metabolism pathways in ALS are warranted.

### 3.3. Mechanism of Action of Edaravone on the Neurotoxicity Induced by TDP-43 and Ethacrynic Acid

In 1464R neuronal cells, TDP-43 and oxidative stress induced the expression of genes related to multiple pathways, including Nrf2, UPR, and autophagy, consistent with previous reports on TDP-43 proteinopathy and ALS [63,64,65]. These changes may contribute to adaptive responses of neuronal cells to oxidative stress and ER stress. Edaravone is presumed to normalize these aberrant pathways in the TDP-43 proteostasis network, leading to the prevention of cell death.

### 3.4. Limitation of the Study

This study has three main limitations. First, this exploratory study was conducted in TDP-43-expressing 1464R neuronal cells as a model of proteinopathy. Confirmatory studies in other ALS/TDP-43 proteinopathy models are considered necessary. Second, this study revealed changes in expression levels of transcripts. Therefore, the impact of these changes in the expression of identified genes and pathways should be analyzed using pharmacological stimulators or inhibitors and gene-manipulated cells/animals. Third, this study used differentiated cells originating from rat neural stem cells. The effects of edaravone on endogenous and spontaneous TDP-43 pathology should be addressed in the future using neuronal cells derived from induced pluripotent stem cells or neural stem cells of human origin harboring mutations associated with ALS.

## 4. Materials and Methods

### 4.1. Adult Rat Neural Stem Cell Line 1464R

All experiments were performed in accordance with the Japanese National Guidelines and Regulations. They were approved by the Biosafety Committee and Animal Care and Use Committee of the Kyorin University Faculty of Health Sciences.

The 1464R adult rat neural stem cell line was established as previously described [12,13]. The cells were cultured in Neurobasal medium (#21103-049; Thermo Fisher Scientific, Waltham, CA, USA) on 10 cm dishes coated with poly-2-hydroxyethyl methacrylate (#P3932; Sigma) to prevent cell attachment in a 5% CO_2_ atmosphere at 37 °C. The growth medium contained 2 mM L-glutamine (#25030-081; Thermo Fisher Scientific), 2% B-27 supplement (#17504-044; Thermo Fisher Scientific), 10 ng/mL fibroblast growth factor 2 (FGF2) (#F0291; Sigma, St. Louis, MO, USA), 10 ng/mL epidermal growth factor (#E9644; Sigma), 50 units/mL penicillin, and 50 μg/mL streptomycin (#15070-063; Thermo Fisher). The 1464R cells, which form typical neurospheres, were mechanically dissociated and serially passaged in the same growth medium twice a week. To differentiate 1464R cells into neuronal and glial cells, the dissociated cells were seeded onto poly-L-lysine (PLL) (#P1524; Sigma)-coated 96-well plates at a density of 8 × 10^4^ cells/well for cell viability assay or 6-well plates at a density of 1 × 10^6^ cells/well for DEG assay, and then maintained in a differentiation medium consisting of F-12 medium (#11765-054; Thermo Fisher Scientific) containing 5% fetal bovine serum (Moregate, Australia), 0.5% N-2 supplement (#17502-048; Thermo Fisher Scientific), 1% B-27 supplement, 1 μmol/L ATRA (#R2625; Sigma), 50 units/mL penicillin, and 50 μg/mL streptomycin in a 5% CO_2_ atmosphere at 37 °C for 4 days.

### 4.2. Adenovirus Infection

For adenovirus infection, the differentiation medium was replaced by a serum-free, antioxidant-free F-12 medium containing 0.5% N-2 supplement, 1 μmol/L ATRA, 50 units/mL penicillin, and 50 μg/mL streptomycin in a 5% CO_2_ atmosphere at 37 °C. Recombinant adenovirus vectors encoding DsRed-tagged full-length human WT (AxDsRhWTTDP43; RIKEN DNA Bank Japan; #RDB15499) and CTF (208-414aa of TDP-43) (AxDsRhCTFTDP43; RIKEN #RDB15500) TDP-43 cDNAs were prepared as described previously [12,13]. The cells on PLL-coated 6- or 96-well plates were infected with adenoviruses at a multiplicity of infection of 50.

### 4.3. Edaravone and/or Ethacrynic Acid Treatment

Edaravone synthesized at Mitsubishi Tanabe Pharma Corporation (Osaka Japan) was added to the cells infected with adenoviruses expressing the WT and CTF TDP-43 at final concentrations of 1–200 μmol/L in the cell viability assay and 50 μmol/L in the sequence analysis assay. Edaravone was prepared in DMSO (100%), and the final concentration of DMSO was set at 0.1% in both vehicle and edaravone treatments. Twenty-four hours later, the cells received a medium either containing or not containing 20 μmol/L ethacrynic acid (#SML1083; Sigma) and were further incubated for 24 h.

### 4.4. Cell Viability Assay

The cells were then incubated with a CCK-8 solution (#CK04; DOJINDO, Tokyo, Japan) at 10 μL/200 μL culture medium for 4 h, and the absorbance at 450 nm was measured with a microplate reader.

### 4.5. Double-Stranded cDNA Amplification

From the frozen pellets of the cultured cells (>10^6^ cells), total RNA was extracted with NucleoSpin RNA (Macherey-Nagel GmbH & Co. KG, Düren, Germany) according to the manufacturer’s instructions. The RNA was of high quality, with an RNA integrity number score of ≥7, RNA concentration of ≥2 ng/µL, and total RNA amount of ≥50 ng, as assessed using a TapeStation and Bioanalyzer RNA 6000 Nano Chip (Agilent Technologies, Inc., Santa Clara, CA, USA).

Using the RNA (1.0 ng) as a template, double-stranded cDNA was synthesized using a Switching Mechanism At 5′ End of RNA Template (SMART) method with SMART-Seq^®^ v4 Ultra^®^ Low Input RNA Kit for Sequencing (Clontech Laboratories, Inc., Mountain View, CA, USA) according to the manufacturer’s instructions. The double-stranded cDNA was amplified in 13 cycles of polymerase chain reaction (PCR). The PCR product was purified using the magnetic bead method with AM Pure XP (Beckman Coulter, Inc., Brea, CA, USA).

### 4.6. Library Preparation

The double-stranded cDNA was fragmented by a tagmentation reaction involving transposon-cleaving and tagging. Subsequently, both ends of the fragmented cDNA were ligated using adapter sequences with the Nextera XT DNA Library Prep Kit and Nextera XT Index Kit v2 (Illumina, Inc., San Diego, CA, USA). The ligated cDNA (0.2 ng) was amplified in 12 cycles of PCR using a unique sequence for each sample. The PCR products from all samples were pooled into one library to avoid sequencing batch effects in the following sequencing step.

### 4.7. Sequencing

The library was sequenced on the NovaSeq 6000 system with NovaSeq 6000 S4 Reagent Kit and NovaSeq Xp 4-Lane Kit (Illumina, Inc., San Diego, CA, USA) using the NovaSeq Control Software (version 1.6.0; Illumina, Inc., San Diego, CA, USA). Base calls and quality scores were generated by Real Time Analysis (version 3.4.4; Illumina, Inc., San Diego, CA, USA). The base call files were converted into FASTQ files using bcl2fastq2 (version 2.20; Illumina, Inc., San Diego, CA, USA).

### 4.8. Sequence Analysis

The resultant 150 base pair-end reads were aligned using the rat genome assembly (version 6.0, top level) and transcript annotation (release 101), downloaded from the Ensembl database [66], on a DRAGEN Bio-IT platform (version 3.6.3; Illumina, Inc., San Diego, CA, USA) using default parameters. On the basis of the alignment to the references, RNA abundance at the gene level was calculated as count and transcript per million (TPM). Note that the TPM per transcript *i* is calculated as follows:

(1) $$TPM_{i}=10^{6}*\frac{m_{i}/l_{i}}{\sum_{i}\left(m_{i}/l_{i}\right)\;},$$

where *m_i_* and *l_i_* represent the number of reads mapped to transcript *i* and length of transcript *i*, respectively. Downstream analyses were performed using R (version 3.6.0) [67].

### 4.9. Principal Component Analysis

For quality check of the RNA sequencing data, principal component analysis was performed with the obtained TPM values after trimming the genes with a variance < 1 among all samples.

### 4.10. Differential Expression Analysis

Using DESeq2 with the count data [15], shrunken log2 fold changes and *p*-values from a Wald test were calculated by adjusting for multiple testing with the BH procedure [16]. Genes were considered statistically significant when meeting the following criteria: (i) absolute log2 fold change ≥ 1 and (ii) adjusted *p*-value ≤ 0.05.

### 4.11. GO Enrichment Analysis

GO data were retrieved from the BioMart database [68,69]. To identify GO terms related to DEGs, a GO enrichment analysis was performed using a hypergeometric distribution to determine which GO terms were significantly overrepresented in the genes compared to what is expected by chance. The resulting *p*-values were adjusted for multiple testing using the BH procedure [16].

### 4.12. qRT-PCRStatistical Analysis

Reverse transcription was performed using SuperScript^®^ VILO^®^ Master Mix (#11755-250, Thermo Fisher Scientific). qRT-PCR was performed using TaqMan^®^ FAST Advanced Master Mix (#4444557, Thermo Fisher Scientific). Relative expression of each gene was normalized to actin beta. The primer sequences (Thermo Fisher Scientific) are listed in Supplemental Table S3.

### 4.13. Pathway Analysis

Pathway analysis was performed using Ingenuity Pathway Analysis (QIAGEN, Inc., Hilden, Germany).

### 4.14. Statistical Analysis

Statistical differences were analyzed using SAS (version 9.4, SAS Institute., Cary, NC, USA) for group comparisons. Two-tailed *p*-value < 0.05 was considered statistically significant.

## 5. Conclusions

Edaravone suppressed neurotoxicity in 1464R-differentiated neuronal cells transduced with adenoviruses expressing WT and CTF TDP-43 and treated with ethacrynic acid. Transcriptome analysis revealed that the protective effects were associated with changes of gene expression in pathways of Nrf2-mediated oxidative stress response, UPR, and autophagy. Furthermore, genes involved in cellular membrane lipid-associated pathways, including inositol phosphate metabolism, as well as 3-phosphoinositide biosynthesis and degradation, were significantly expressed upon edaravone treatment. Thus, the present findings identify novel putative molecular target pathways underlying edaravone treatment in sporadic ALS patients with TDP-43 proteinopathy.

## Figures and Tables

**Table 1:** Number of differentially expressed genes.

Group	Group 1	Group 2	Group 3	Group 4
Group 1 (Control)		0/0	853/467	1654/1095
Group 2 (Eda)			876/478	1661/1130
Group 3 (TDP-43 + EA)				169/260
Group 4 (TDP-43 + EA + Eda)				

Group 1, Control; Group 2, edaravone-treated cells (Eda); Group 3, ethacrynic acid-treated cells expressing TDP-43 (TDP43 + EA); and Group 4, edaravone- and ethacrynic acid-treated cells expressing TDP-43 (TDP43 + EA + Eda). The numerator and denominator values in each column represent the number of genes that were up- or downregulated in the corresponding column group compared to the corresponding row group, respectively. Eda—Edaravone; EA—Ethacrynic acid.

**Table 2:** List of representative genes whose expression was significantly changed by edaravone treatment.

Gene Name	Description	Group 3 to Group 1		Group 4 to Group 3	
		log2 Ratio	*p* Values	log2 Ratio	*p* Values
**Upregulated genes ^a^**					
Ho-1	heme oxygenase 1	6.601	0.000	1.755	4.712 × 10^−44^
Cebpb	CCAAT/enhancer binding protein beta	2.930	4.358 × 10^−164^	1.661	1.309 × 10^−28^
Plpp3	phospholipid phosphatase 3	0.751	7.027 × 10^−4^	1.307	3.287 × 10^−10^
Sqstm1	sequestosome 1	1.366	1.227 × 10^−55^	1.225	2.665 × 10^−26^
Pik3cb	phosphatidylinositol-4,5-bisphosphate 3-kinase, catalytic subunit beta	0.879	8.668 × 10^−11^	1.157	2.285 × 10^−18^
Txndc2	thioredoxin domain-containing 2	0.917	4.981 × 10^−2^	1.153	5.690 × 10^−3^
Hspa8	heat shock protein family A (Hsp70) member 8	1.931	1.714 × 10^−79^	1.151	4.385 × 10^−13^
Hsph1	heat shock protein family H (Hsp110) member 1	4.000	0.000	1.027	2.124 × 10^−7^
Ptgds	prostaglandin D2 synthase	−1.115	1.478 × 10^−4^	0.954	2.334 × 10^−3^
Nfe2l2	nuclear factor erythroid 2-like 2	1.898	1.063 × 10^−43^	0.923	9.336 × 10^−11^
Plcg2	phospholipase C, gamma 2	−1.570	3.015 × 10^−11^	0.881	4.049 × 10^−3^
**Downregulated genes ^b^**					
Nefl	neurofilament light	0.738	1.168 × 10^−1^	−1.778	5.902 × 10^−7^
Acsl6	acyl-CoA synthetase long-chain family member 6	−1.225	2.073 × 10^−6^	−1.582	1.693 × 10^−5^
Mt2A	metallothionein 2A	2.134	5.863 × 10^−9^	−1.425	9.110 × 10^−5^
Mt1	metallothionein 1	0.866	8.031 × 10^−6^	−1.392	3.600 × 10^−9^
Glrx	glutaredoxin	0.726	3.319 × 10^−3^	−1.262	8.634 × 10^−6^
Alpl	alkaline phosphatase, biomineralization associated	1.344	1.392 × 10^−4^	−1.098	1.057 × 10^−3^
Nup43	nucleoporin 43	0.947	6.178 × 10^−5^	−0.888	1.606 × 10^−4^

^a^ Upregulated significantly (adjusted *p*-value ≤ 0.01 and/or log2 fold change ≥ 1) in Group 4 compared to Group 3. ^b^ Downregulated significantly (adjusted p-value ≤ 0.01 and/or log2 fold change ≤ −1) in Group 4 compared to Group 3.

## Data Availability

The FASTQ files are supposed to be deposited in the Gene Expression Omnibus archive at the National Center for Biotechnology Information (GSE207578; https://www.ncbi.nlm.nih.gov/geo/query/acc.cgi?acc=GSE207578 (accessed on 6 July 2022)) [70,71].

## Supplementary Materials

The following are available online at https://www.mdpi.com/article/10.3390/ph15070842/s1. Figure S1. Effects of edaravone (simultaneous treatment with ethacrynic acid for 24 h) against neurotoxicity in cells transduced with adenoviruses expressing WT and CTF TDP-43; Figure S2. Cell viability of 1464R-derived cells expressing TDP-43 for 48 h and the effect of ethacrynic acid (EA) treatment for 24 h following expression of TDP-43; Figure S3. Effects of edaravone on TDP43-expression-induced neurotoxicity without ethacrynic acid in rat neural stem cell-derived neurons; Figure S4. The heatmap of the number of DEGs by inter-group comparative analysis to all combinations; Figure S5. GO enrichment analysis on pharmacological effects of edaravone and/or ethacrynic acid (EA) treatment in TDP-43 expressed and normal cells; Figure S6. GO enrichment analysis among differentiated neuronal cells affected by neurotoxicity induced by ethacrynic acid; Table S1. The comparison of gene expression profiles between cells TDP-43 expressing and non-expressing (control) cells; Table S2. Expression levels of genes related to PI metabolism pathways between the Control (Group 1) and the TDP43+EA groups (Group 3); Table S3. List of qPCR primers used in experiments in Figure 6.
