# Supplementary material for: The Protective Effect of Edaravone on TDP-43 Plus Oxidative Stress-Induced Neurotoxicity in Neuronal Cells: Analysis of Its Neuroprotective Mechanisms Using RNA Sequencing
Source: Pharmaceuticals (Basel). 2022 Jul 8;15(7):842. doi: 10.3390/ph15070842 (PMC9319738; doi:10.3390/ph15070842)
Supplement: Supplementary file 1 [file pharmaceuticals-15-00842-s001.zip › pharmaceuticals-1783734-supplementary.pdf]

Supplemental Data

The protective effect of edaravone on TDP-43 plus oxidative stress-induced neurotoxicity in neuronal cells: Analysis of its neuroprotective mechanisms using RNA sequencing

Aki Soejima-Kusunoki [1,*], Kinya Okada [2], Ryuta Saito [2] and Kazuhiko Watabe [3]

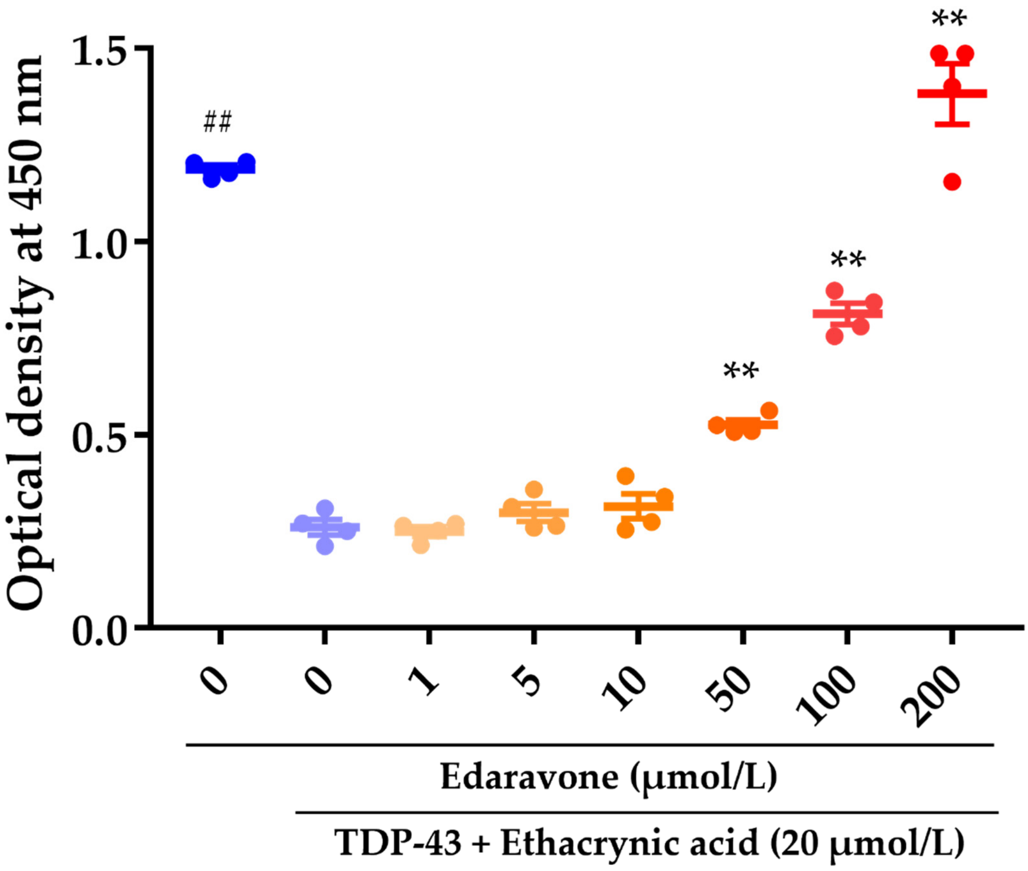

Figure S1. Effects of edaravone (simultaneous treatment with etacrynic acid for 24 h) against neurotoxicity in cells transduced with adenoviruses expressing WT and CTF TDP-43

Neurotoxicity was analyzed as a decrease in cell viability using the CCK-8 assay. Data are expressed as mean ± standard error of the mean (SEM) (n = 4 wells in 1 experiment). ##p < 0.01 compared to

the group of TDP-43 plus ethacrynic acid (Student t-test). **$p < 0.01$ compared to the group of TDP-43 plus ethacrynic acid (Williams' multiple comparison test).

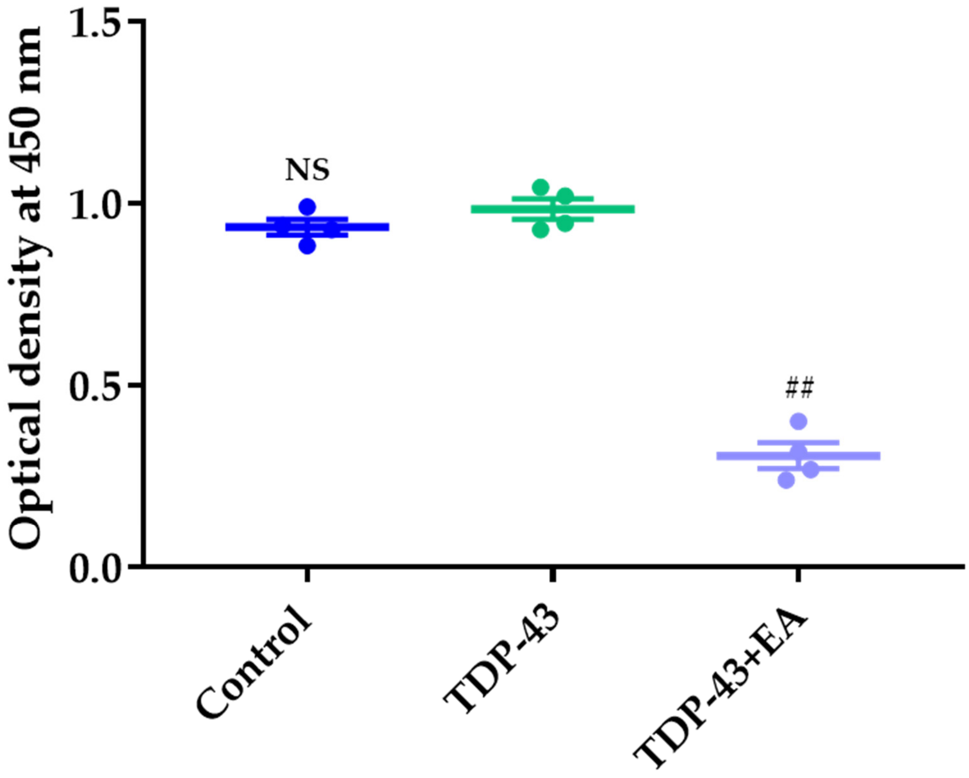

Figure S2. Cell viability of 1464R-derived cells expressing TDP-43 for 48 h and the effect of etacrynic acid (EA) treatment for 24 h following expression of TDP-43

Data expressed as mean ± SEM (N = 4). ##P < 0.01; NS: Not significant vs. TDP-43 (t-test).

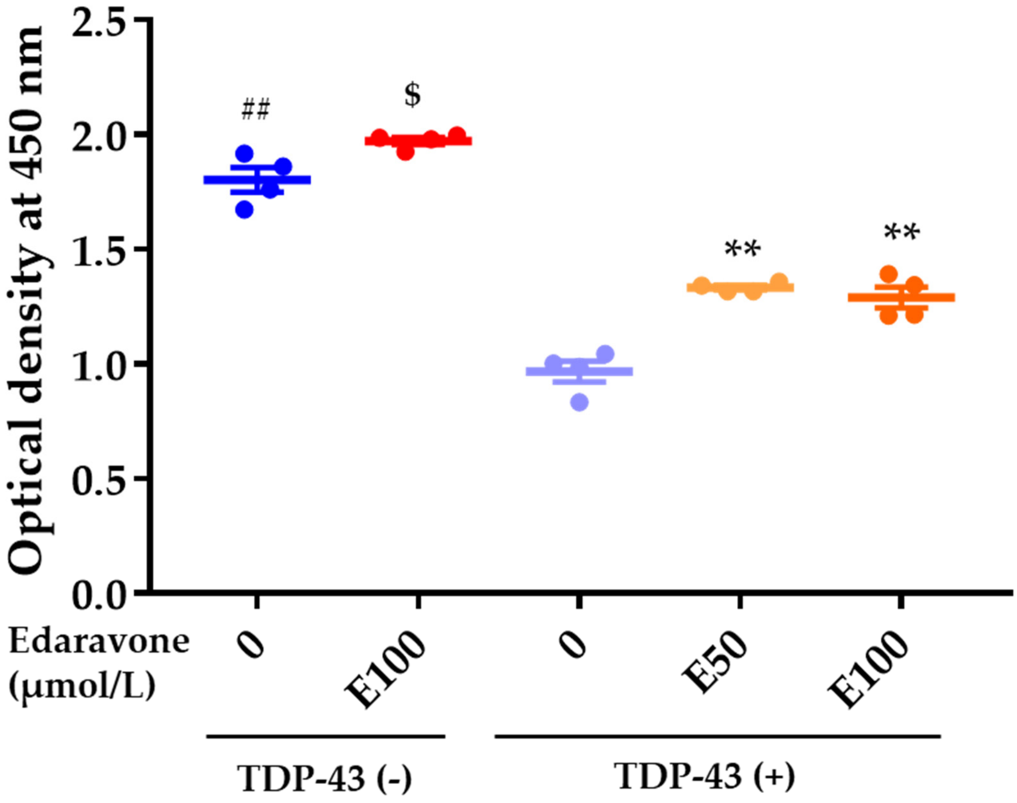

Figure S3. Effects of edaravone on TDP43-expression-induced neurotoxicity without ethacrynic acid in rat neural stem cell-derived neurons

Normal or TDP-43-expressed cells were maintained for 48 h, and edaravone at various concentrations was treated for another 48 h. The neurotoxicity was analyzed as a decrease in cell viability with the CCK-8 assay. Data expressed as mean ± SEM (N = 4). ##P < 0.01 vs. TDP43 (+) control (t-test); **P < 0.01 vs. TDP43 (+) control (Williams' multiple comparison test); $p < 0.05 vs. TDP-43 (−) control (t-test).

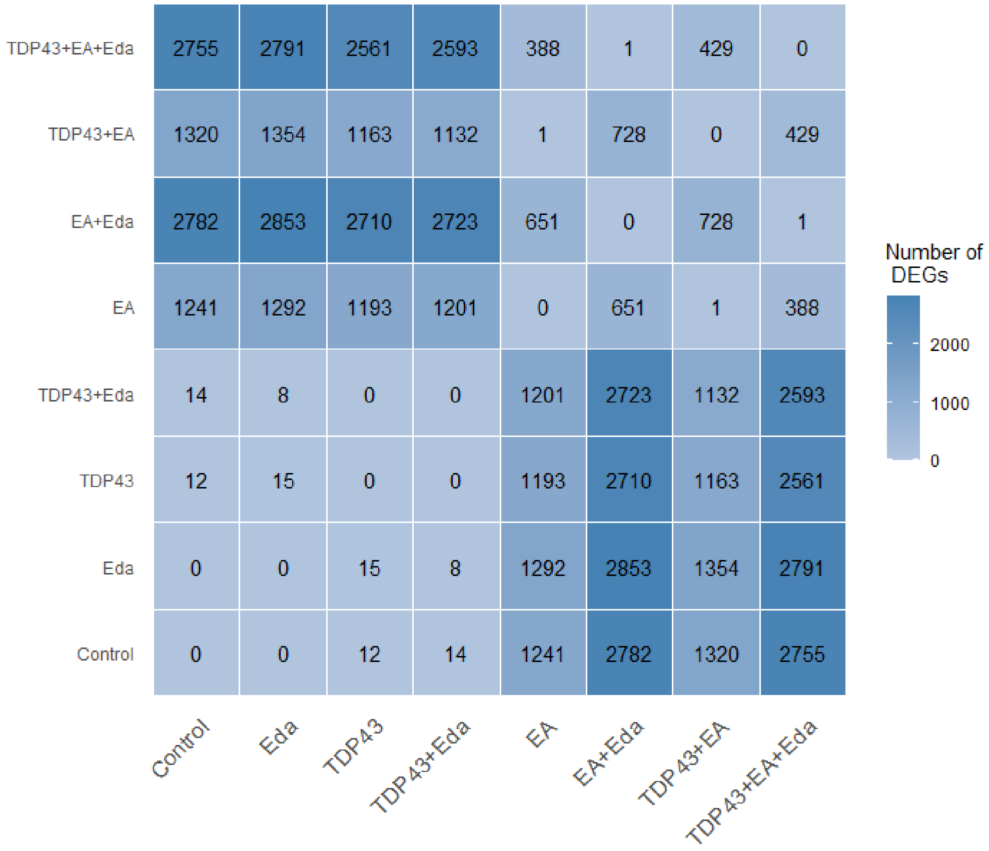

Figure S4. The heatmap of the number of DEGs by inter-group comparative analysis to all combinations

EA, ethacrynic acid; Eda, Edaravone; TDP-43, TDP-43-expressed cells.

The number of DEGs in normal control vs. TDP-43-expressed, normal control vs normal control + EA, normal control vs. TDP-43-expressed+ EA, TDP-43-expressed vs. TDP-43-expressed + EA was 12, 1241, 1320, and 1163, respectively.

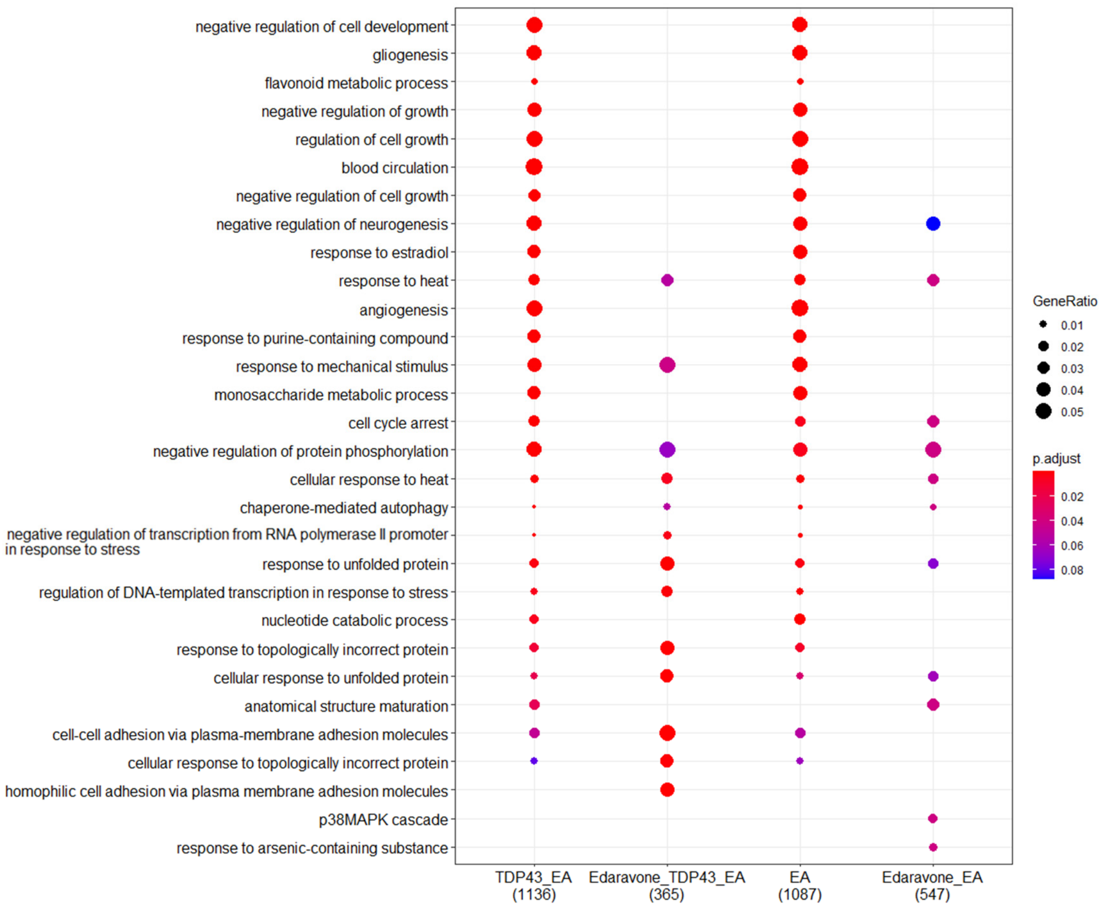

Figure S5. GO enrichment analysis about pharmacological effects of edaravone and/or ethacrynic acid (EA) treatment in TDP-43 expressed and normal cells

DEGs of edaravone treatment were enriched in similar GO categories in EA-treated normal and TDP-43-expressed cells.

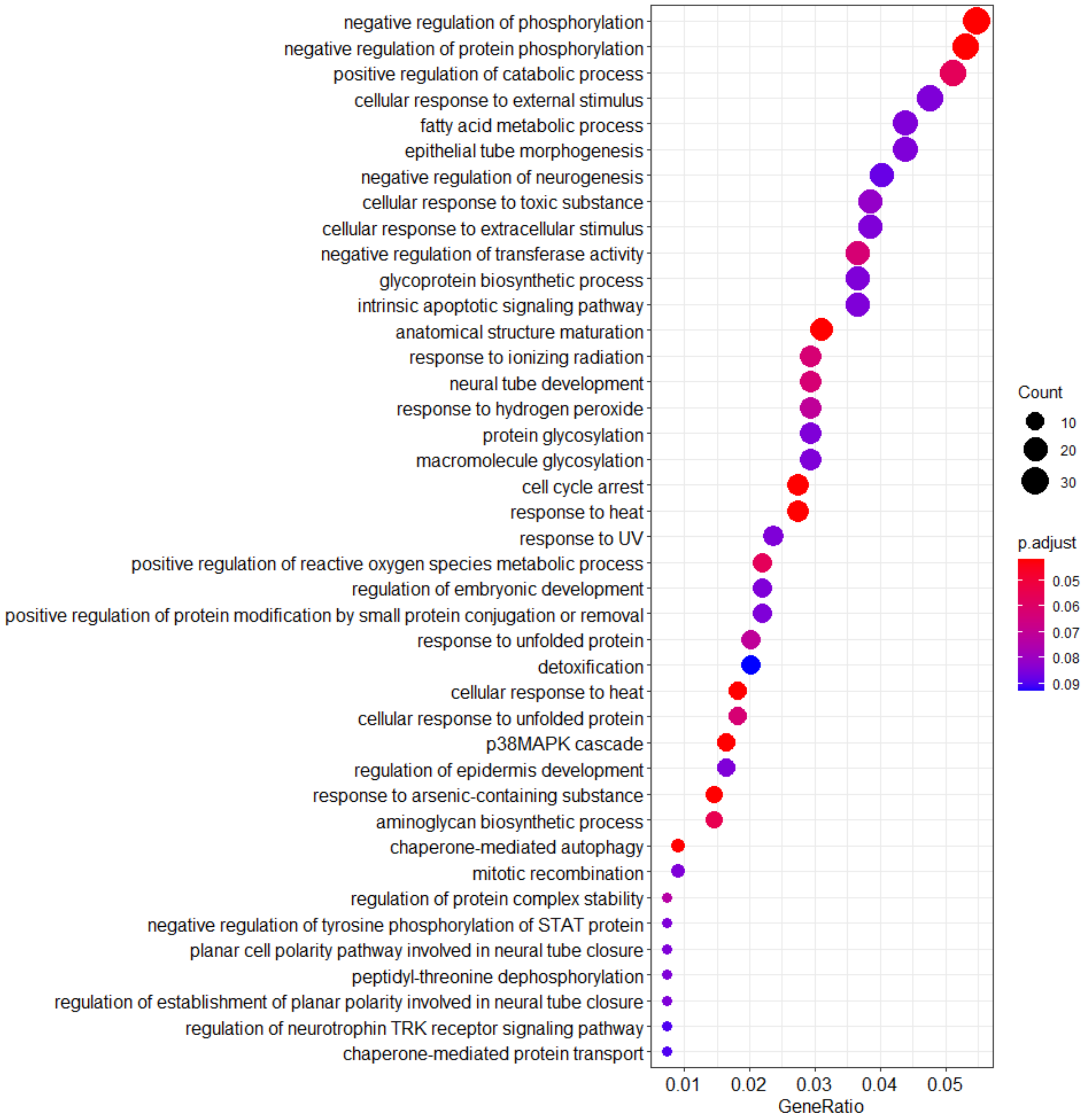

Figure S6. GO enrichment analysis among differentiated neuronal cells affected by neurotoxicity induced by ethacrynic acid

The enriched pathways were identified by p-value adjusted with the BH procedure < 0.1 and ranked DEGs gene ratio in GO categories.

Table S1. The comparison of gene expression profiles between cells TDP-43 expressing and non-expressing (control) cells

Gene Name	Description	q-value	log2 fold-change
Nusap1	nucleolar and spindle associated protein 1 [Source:RGD Symbol;Acc:1305764]	1.36E-14	1.12E+00
Ninl	ninein-like [Source:RGD Symbol;Acc:1306152]	8.95E-25	1.30E+00
Tardbp	TAR DNA binding protein [Source:RGD Symbol;Acc:1310906]	0.00E+00	4.06E+00
Tnfsf9	TNF superfamily member 9 [Source:RGD Symbol;Acc:727974]	9.26E-14	1.04E+00
Dok7	docking protein 7 [Source:RGD Symbol;Acc:1566416]	4.51E-24	1.44E+00
Fam161a	FAM161 centrosomal protein A [Source:RGD Symbol;Acc:1304999]	2.14E-14	1.03E+00
Ccnf	cyclin F [Source:RGD Symbol;Acc:67401]	2.51E-14	1.05E+00
Tnfrsf19	TNF receptor superfamily member 19 [Source:RGD Symbol;Acc:1564996]	1.12E-50	1.30E+00
Serpinb1a	serpin family B member 1A [Source:RGD Symbol;Acc:1306203]	4.48E-12	1.03E+00
Vgf	VGF nerve growth factor inducible [Source:RGD Symbol;Acc:69399]	2.47E-12	1.05E+00
Mmp11	matrix metallopeptidase 11 [Source:RGD Symbol;Acc:3099]	3.40E-13	1.02E+00

Table S2. Expression levels of genes related to PI metabolism pathways between the Control (Group 1) and the TDP43+EA groups (Group 3)

Ingenuity Canonical Pathways	-log(B-H p-value)	log2 Ratio
3-phosphoinositide Degradation	2.05E+00	1.11E-01
D-myo-inositol-5-phosphate Metabolism	1.95E+00	1.09E-01
Superpathway of Inositol Phosphate Compounds	1.95E+00	1.03E-01
D-myo-inositol (1,4,5,6)-Tetrakisphosphate Biosynthesis	1.77E+00	1.07E-01
D-myo-inositol (3,4,5,6)-tetrakisphosphate Biosynthesis	1.77E+00	1.07E-01
3-phosphoinositide Biosynthesis	1.54E+00	9.85E-02

Table S3. List of qPCR primers used in experiments in Figure 6

Hmox1(Ho-1)_Rn00561387_m1
Sqstm1_Rn00709977_m1
Pik3cb_Rn00585107_m1
Nfe2l2_Rn00582415_m1
Plcg2_Rn01431998_m1
Stip1_Rn01647759_g1
Nefl_Rn00582365_m1
Acsl6_Rn00590587_m1
Mt2A,Mt1m_Rn01536588_gH
Glrx_Rn00574074_m1
Alpl_Rn01516028_m1
Nup43_Rn01440850_m1
Actb_Rn00667869_m1
